# Skp2 Regulates Subcellular Localization of PPARγ by MEK Signaling Pathways in Human Breast Cancer

**DOI:** 10.3390/ijms140816554

**Published:** 2013-08-09

**Authors:** Hongge Cheng, Jie Meng, Guisheng Wang, Yuming Meng, Yu Li, Dong Wei, Chunyun Fu, Kaifeng Deng, Aiguo Shen, Huimin Wang, Shengming Dai

**Affiliations:** 1 Department of Laboratory Science, the Fourth Hospital Affiliated to Guangxi Medical University, Liuzhou 545005, Guangxi, China; E-Mails: ge_ge_bu_ru@sina.com (H.C.); mengjie840822@163.com (J.M.); wangguisheng@china.com (G.W.); mengyuming1@sina.com (Y.M.); lgzhqsh@163.com (Y.L.); weidong415@126.com (D.W.); fuchunyun2008@sina.com (C.F.); lgjyk2001@163.com (K.D.); 2 Department of Immunology and Microbiology, Medical College of Nantong University, Nantong 226001, Jiangsu, China; E-Mail: shag@ntu.edu.cn; 3 Medical Laboratory Center, Affiliated Hospital of Nantong University, Nantong 226001, Jiangsu, China

**Keywords:** cytoplasmic localization, peroxisome proliferator-activated receptors γ (PPARγ), . S-phase kinase protein (Skp2), human breast cancer

## Abstract

Nuclear hormone receptor family member PPARγ plays an important role in mammary gland tumorigenesis. Previous studies have shown PPARγ has cytoplasmic activities upon tetradecanoyl phorbol acetate (TPA) stimulation. However, the clinical pathological significance of cytoplasmic PPARγ is not completely understood in human breast cancer. Skp2 is oncogenic, and its frequent amplification and overexpression correlated with the grade of malignancy. In this study, the role of cytoplasmic PPARγ and Skp2 expression was investigated in human breast cancer progression. Therefore, immunohistochemical analysis was performed on formalin-fixed paraffin sections of 70 specimens. Furthermore, Western blot and immunofluorescence microscopy analysis were used to study the relationship between expression of cytoplasmic PPARγ and Skp2 expression in human breast cancer cells *in vitro*. Results showed that the expression of cytoplasmic PPARγ was positively correlated with Skp2 expression (*p* < 0.05), and correlated significantly with estrogen receptor (*p* = 0.026) and pathological grade (*p* = 0.029), respectively. In addition, Skp2 overexpression can provoke cytoplasmic localization of PPARγ upon MEK1-dependent mechanisms in human breast cancer cells by nuclear-cytosolic fractionation technology and immunofluorescence microscopy analysis. Using RNA interference technology, we also found that down-regulated Skp2 reduced the phosphorylation level of MEK1 and significantly reversed TPA-induced nuclear export of PPARγ in MDA-MB-231 cells. The changes in the subcellular localization of PPARγ may represent a novel target for selective interference in patients with breast cancer.

## 1. Introduction

Breast cance0r is one of the deadliest cancers and a complex disease that results from a multi-stage process involving the deregulation of a number of different signaling cascades. Anti-estrogen hormone therapy has been best used for prevention and treatment in women with early breast cancer [1]. However, hormone therapy has little effect on estrogen receptor-negative (ER-negative) tumors [2]. Therefore, an evolving understanding of the genetic and molecular alterations in breast cancer has led to the development of novel agents that may contribute to an extension of patient survival and eventually a cure for this devastating malignancy.

Peroxisome proliferator-activated receptors (PPAR) are ligand-activated transcription factors belonging to the nuclear hormone receptor family [3,4]. Among the three PPAR isoforms (PPARα, β/δ, and γ), PPARγ influences biological processes such as inflammation, cell survival, differentiation, cell proliferation and tumorigenesis [4,5]. 15-Deoxy-D12,14-prostaglandin J2(15D-PGJ2) is a natural ligand for PPARγ and thiazolidinediones (troglitazone as an example) are synthetic ligands for PPARγ. PPARγ is highly expressed in fatty tissues and many human cancers, including breast cancer [6]. Moreover, PPARγ has diverse biological functions, such as promoting terminal differentiation of adipocytes, inducing differentiation and apoptosis of tumor cells and inhibiting tumor angiogenesis. These mechanisms require nuclear PPARγ localization, but recent data provided evidence that PPARγ also localized to the cytoplasm. Little is known about the mechanisms that provoke nucleocytoplasmic shuttling of PPARγ. Recently, Burgermeister [7] reported that PPARγ was shuttled from the nucleus by binding its AF2 domain to MEK1, which increased the level of MEK1 phosphorylation upon TPA stimulation. This complex was actively exported from the nucleus to the cytosol by CRM1 in response to TPA stimulation, thereby causing PPARγ downregulation in the nucleus and inhibiting PPARγ-dependent transactivation [8]. Khateeb *et al.* found that urokinase-type plasminogen activator can stimulate PPARγ nuclear export in hepatocytes, resulting in downregulation of paraoxonase 1 (PON1) expression [9]. However, whether cytoplasmic PPARγ might be important for predicting human breast cancer development and progression was not elucidated.

S-phase kinase protein (Skp2) is oncogenic, and its frequent amplification and overexpression correlates with the grade of malignancy in certain tumors. Skp2 is the specific substrate-recognition subunit of the Skp1-Cullin-F-box protein (SCF) type ubiquitin ligase complex [10], which mediates ubiquitin-dependent degradation of some cell-cycle proteins and transcription factors, such as p27, p130, myc, and p57 [11]. Skp2 plays an important role in breast cancer, and is also considered to have strong independent prognostic potential. Signoretti *et al.* have recently showed that Skp2 was overexpressed in either the primary and local recurrences or metastatic breast cancers that were negative for both estrogen receptor and HER-2 receptor [12,13]. A relationship between Skp2 expression level and a poor prognosis was also observed, for example, in B-cell lymphoma [14], and breast cancer [15]. Our group has previously shown that overexpression of PPARγ can down-regulate Skp2 expression in MDA-MB-231 breast tumor cells [16], but whether or not Skp2 influences PPARγ function in breast cancer has not yet been determined.

In this study, we aim to study the relationship between expression of cytoplasmic PPARγ and Skp2 expression in breast cancer, and investigate the mechanisms by which Skp2 regulates cytoplasmic localization of PPARγ. Because Skp2 is located downstream in the MAPK signaling pathway, we have in the present study tested the hypothesis that Skp2 overexpression can provoke cytoplasmic localization of PPARγ upon MEK1-dependent mechanisms in human breast cancer cells. Our results emphasize the importance of the cytoplasmic localization of PPARγ in the development and progression of breast cancer. The findings here also support a therapeutic target in this pathway for treating breast cancer.

## 2. Results

### 2.1. The Expression of Cytoplasmic PPARγ Is Positively Related with Skp2 Expression

**Figure 1:**
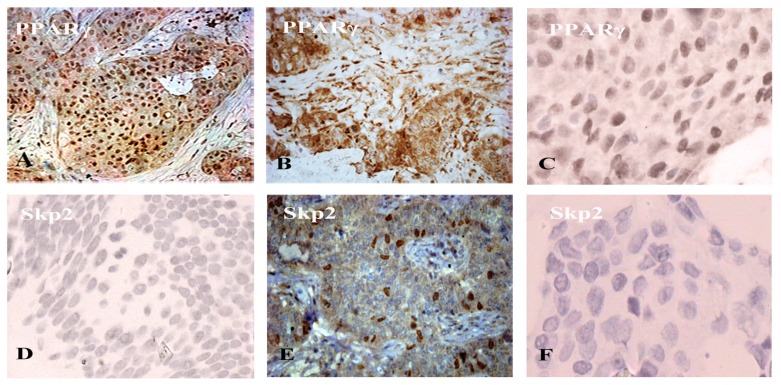
Representative immunohistochemistry slides of Skp2 and PPARγ staining in locally advanced breast cancer. PPARγ and Skp2 protein expression in paraffin-embedded human breast benign and malignant tumors (×400). (**A**–**C**) PPARγ reactivity; **(D**–**F**) Corresponding Skp2 staining. (**A**) Benign breast disease sample displaying strong nuclear staining for PPARγ; (**B**) Infiltrating ductal carcinoma (ER-negative, PR-negative, and HER-2-negative, Grade III) showing strong cytoplasmic PPARγ immunostaining; (**C**) ER-positive human breast cancer sample (Grade I) showed reducing cytoplasmic PPARγ staining; (**D**) Benign breast disease sample displaying weak Skp2 immunostaining; (**E**) Infiltrating ductal carcinoma (ER-negative, PR-negative, and HER-2-negative, Grade III) showing strong Skp2 staining; (**F**) ER-positive human breast cancer sample (Grade I) showed weak Skp2 abundance.

Using immunohistochemistry, we examined the clinical significance of cytoplasmic PPARγ and Skp2 in paraffin-embedded mammary tissue sections screened from twenty benign breast diseases and fifty breast cancer patients. We found that PPARγ in the benign breast disease showed positive immunoreactivity mostly in the nucleus (Figure 1A), while ER-negative breast carcinoma samples (poorly differentiated) displayed cytoplasmic mainly staining (Figure 1B). To study the pathological significance of cytoplasmic PPARγ in human breast cancer, the expression of PPARγ was independently evaluated in the cytoplasm in this study. The typical case showed that the high expression of cytoplasmic PPARγ was correlated with high Skp2 expression in the same breast cancer specimen (ER-negative, PR-negative, stage III) (Figure 1B,E). The expression of cytoplasmic PPARγ was reduced in estrogen receptor positive (ER-positive) human breast cancer (well differentiated) correlating with weak Skp2 abundance (Figure 1C,F).

To examine the relationship between the expression of cytoplasmic PPARγ and common parameters associated with tumor behavior, we compared cytoplasmic PPARγ levels with the clinicopathological features described in Table 1. A significant correlation was found among expression of cytoplasmic PPARγ, ER expression (*p* = 0.026), and histologic grade (*p* = 0.029). Thus, the high expression of cytoplasmic PPARγ was associated with high histologic grade and negative ER. We did not observe a significant correlation among cytoplasmic PPARγ levels and PR status (*p* = 0.055), HER-2 status (*p* = 0.941), p53 (*p* = 0.164), TNM (*p* = 0.580), and Ki-67 (*p* = 0.304).

### 2.2. Subcellular Localization of PPARγ in Human Breast Cancer Cells

**Figure 2:**
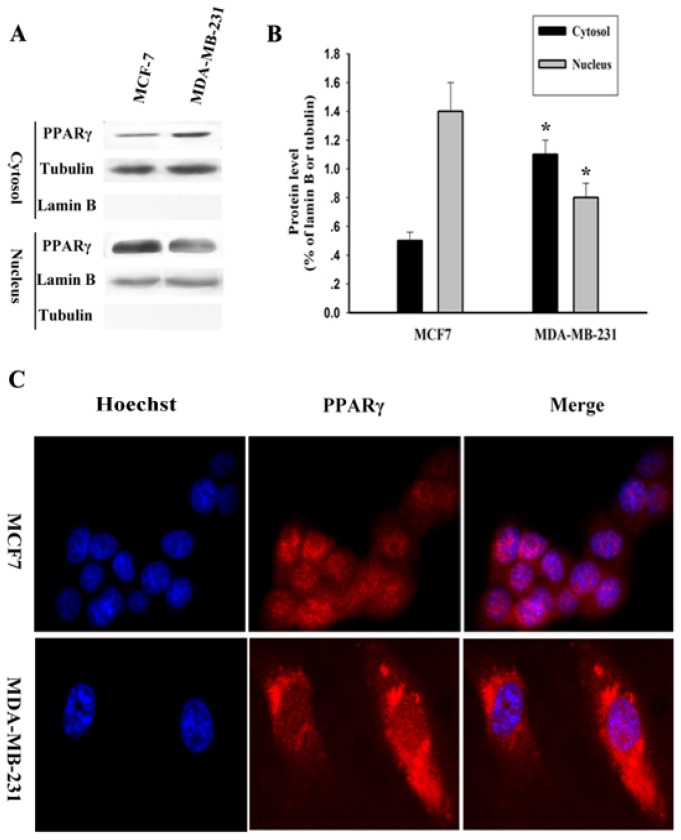
Subcellular localization of PPARγ in human breast cancer cells. (**A**) Equal amounts of cytosolic and nuclear lysates from MCF-7 or MDA-MB-231 cells were analyzed by Western blot to detect PPARγ localization. Lamin B or Tubulin were used as internal controls for nuclear or cytosolic protein, respectively; (**B**) Blot for PPARγ was quantified by densitometry and expression level relative to Lamin B or Tubulin were calculated. The data are means ± SEM (*n* = 3, *p < 0.05, compared with control: MCF-7); (**C**) MCF-7 and MDA-MB-231 cells (1 × 10^5^ cells/well) cultured in 24-well plates separately. 24 h later cells were fixed for detecting the subcellular location of PPARγ by immunofluorescence assay as described in Materials and Methods.

To explore the significance of PPARγ in human breast cancer progression, we examined the subcellular distribution of endogenous PPARγ in human breast cancer cells. Subcellular fractionation revealed PPARγ localized primarily in the nucleus in MCF-7 cell lines (ER-positive), while MDA-MB-231(ER-negative) displayed mainly cytoplasmic localization (Figure 2A,B); thus, MCF-7 cell lines were used in subsequent transfection experiments. Our previous results have showed that MDA-MB-231(ER-negative) which had high invasive capacity displayed high expression levels of Skp2 [16]. Therefore, the relative abundance of cytoplasmic PPARγ and Skp2 appeared to exhibit a positive correlation in MDA-MB-231 cells. Next we used immunofluorescence microscopy technology to further confirm the results shown in Figure 2C. Indeed, we also found subcellular distribution of endogenous PPARγ was association with Skp2 expression.

### 2.3. Cytosolic Retention of PPARγ upon Overexpression of Myc-Skp2

**Figure 3:**
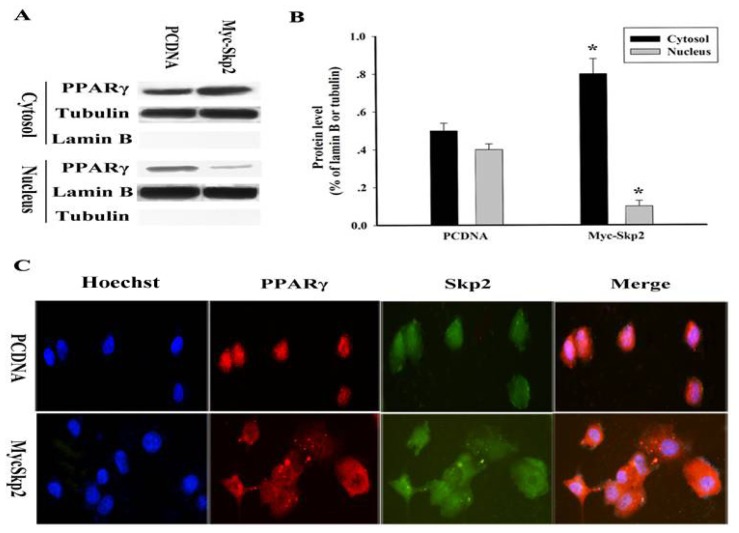
Cytosolic retention of PPARγ upon overexpression of Myc-Skp2. (**A**) MCF-7 cells were transiently transfected with Myc-Skp2 or Myc-empty. Forty-eight hours after transfection, the cells were subjected to cellular fractionation and analyzed by Western blot to detect PPARγ localization; (**B**) Blot for PPARγ was quantified by densitometry and expression level relative to Lamin B or Tubulin were calculated. The data were means ± SEM (*n* = 3, *p < 0.05, compared with Myc-empty: PCDNA); (**C**) MCF-7 cells were grown on coverslips and transiently transfected with either Myc-empty or Myc-Skp2. Forty-eight hours after transfection, the cells were fixed and stained with PPARγ antibodies or Hoechst, and the localization was assessed by fluorescence microscopy as above.

We next sought to determine whether Skp2 can influence subcellular localization of endogenous PPARγ. Thus, MCF-7 cells were transiently transfected with Myc-Skp2 or Myc-empty (PCDNA). Forty-eight hours after transfection, the cells were subjected to cellular fractionation. Using nuclear and cytosolic fractionation technology, a significant decrease of PPARγ in the nucleus was observed in Skp2-transfected cells (Figure 3A). We quantified PPARγ by densitometry and found that cytoplasm-localized PPARγ increased to about 1.6-fold in Skp2-transfected cells as compared to Myc-empty-transfected cells (Figure 3B). Moreover, immunofluorescence analysis also revealed PPARγ was retained in the cytosol in Skp2-transfected cells (Figure 3C).

### 2.4. PPARγ Was Retained in the Cytosol upon MEK1-Dependent Mechanisms in MDA-MB-231 Cells

**Figure 4:**
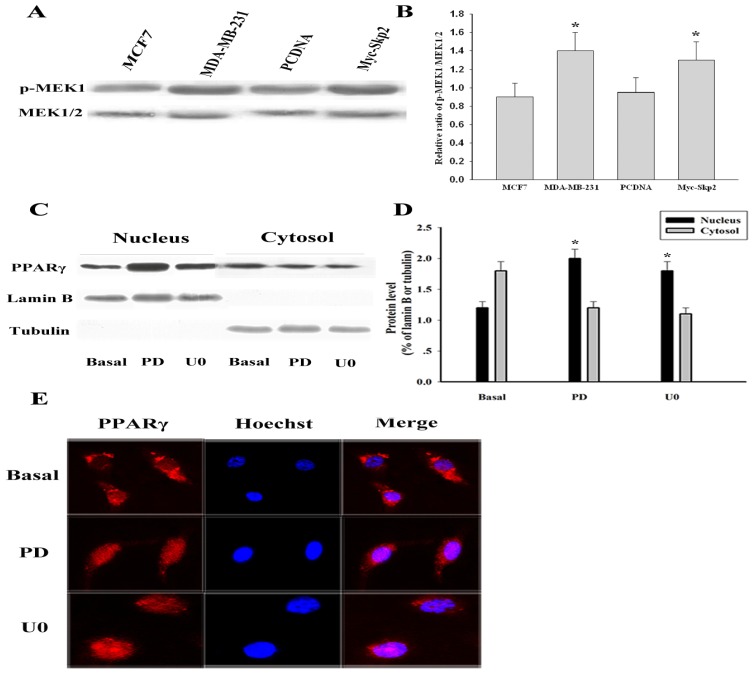
PPARγ was retained in the cytosol upon MEK1-dependent mechanisms. (**A**) Total proteins were extracted and MEK1/2 and their p-MEK1 detected by Western blot in MCF-7 cells, MDA-MB-231 cells, MCF-7 cells transfected with Myc-empty or Myc-Skp2; (**B**) Protein expression was normalized against MEK1/2. Statistical differences compared with the controls were given as *p < 0.05; (**C**) MDA-MB-231 cells were treated with 10 μM of MEK inhibitor PD98059 or U0126 for 12 h, and the cells were subjected to cellular fractionation and blotted with PPARγ antibodies. Control cells were incubated with DMSO vehicle for the same period of time; (**D**) The blot for PPARγ was quantified by densitometry and the expression levels relative to Lamin B or Tubulin were calculated. The data are means ± SEM (*n* = 3, *p < 0.05, compared with control: Basal); (**E**) MDA-MB-231 cells were treated with 10 μM of MEK inhibitor PD98059 or U0126 for 12 h, and the cells were fixed and stained with PPARγ antibodies or Hoechst, and the localization was assessed by fluorescence microscopy as above.

Extracellular signals which activate intracellular phosphorylation pathways can influence the subcellular localization of PPARγ [17]. Previous studies have shown that PPARγ can be phosphorylated by the MEK/ERK signaling pathways [7]. In this study, we found that MDA-MB-231 cell lines displayed higher expression levels of p-MEK1 than MCF-7 cell lines. Moreover, overexpression of Myc-Skp2 resulted in increased MEK1 phosphorylation in MCF-7 cell lines (Figure 4A). Importantly, nuclear localization of PPARγ was induced by the MEK inhibitor PD98059 in MDA-MB-231 cells. We also found pretreatment with U0126 significantly prevented cytoplasmic localization of PPARγ in MDA-MB-231 cells, with a similar effect as PD98059 (Figure 4C). Then, we verified that the relocalization of PPARγ was mediated by MEK signaling pathways by using immunofluorescence techniques (Figure 4E). Our results showed that PPARγ was exported from the cytosol toward the nucleus upon stimulation with PD98059 or U0126 in MDA-MB-231 cells, indicating that MEK1 plays an important role in determining the subcellular localization of PPARγ in human breast cancer.

### 2.5. Down-Regulated Skp2 Reduced Phosphorylation Level of MEK1 and Significantly Reversed TPA-Induced Nuclear Export of PPARγ in MDA-MB-231 Cells

**Figure 5:**
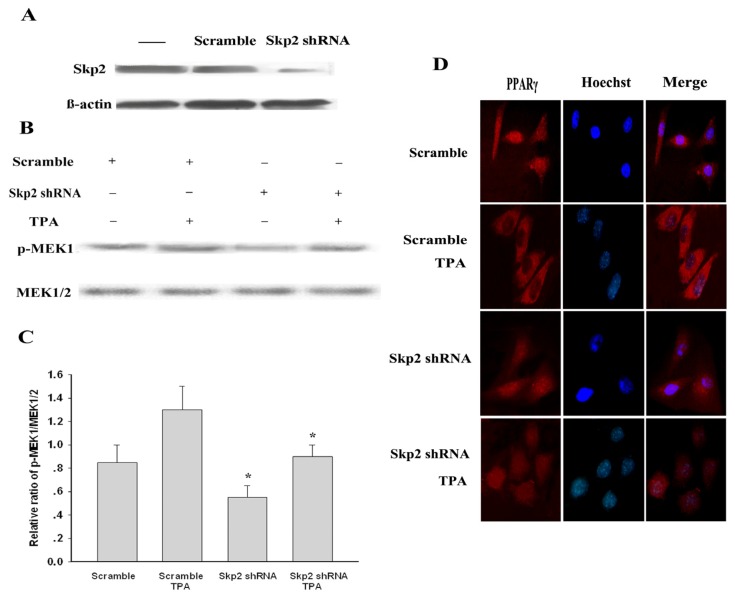
Down-regulated Skp2 significantly reversed TPA-induced nuclear export of PPARγ in MDA-MB-231 cells. (**A**) The cells were transfected with scrambled shRNA or Skp2 shRNA, and expression of Skp2 was evaluated by Western blot analysis; (**B**) MDA-MB-231 cells were transfected with either shRNA oligonucleotides of Skp2 (Skp2 shRNA) or scrambled shRNA (scramble). Forty-eight hours after transfection, the cells were serum starved (0.1% FCS, 16 h) and then were treated with TPA (250 nM, 30 min) or left untreated. Phosphorylation of MEK1 upon TPA stimulation of Skp2-knockdown cells was determined by pMEK and MEK_1/2_ Abs; (**C**) Protein expression was normalized against MEK1/2. Statistical differences compared with the controls were given as *p < 0.05; (**D**) MDA-MB-231 cells were transfected with either Skp2 shRNA or scrambled shRNA and then grown on coverslips as described above. Forty-eight hours after transfection, the cells were serum starved (0.1% FCS, 16 h) and then were treated with TPA (250 nM, 30 min) or left untreated. The cells were stained with polyclonal rabbit PPARγ and Hoechst and developed with Cy3-conjugated anti-rabbit secondary Ab. The localization of PPARγ was visualized by a fluorescence microscopy as above.

To futher verify the above-mentioned results, a Skp2-shRNA expression plasmid was constructed to examine the role of Skp2 on the subcellular localization of endogenous PPARγ. MDA-MB-231 cells were transiently transfected with Skp2-shRNA or scrambled shRNA, and the transfection efficiency was evaluated by Western blots after 48 h (Figure 5A). Then, we investigated the phosphorylation level of MEK1 in Skp2 shRNA-transfected cells. As shown in Figure 5B, Skp2 knockdown reduced the phosphorylation level of MEK1 in MDA-MB-231 cells compared with scrambled shRNA-transfected cells. Previous studies have shown that PPARγ has cytoplasmic activities upon TPA stimulation; we further investigated the relationship of TPA and Skp2-shRNA. MDA-MB-231 cells were treated with 250 nM TPA and Skp2-shRNA alone or in combination for 30 min. Interestingly, the combination of Skp2-shRNA with 250 nM TPA reduced the expression levels of endogenous MEK1 by about 35% compared with TPA-treated cells (Figure 5C). Moreover, we found that the subcellular distribution of endogenous PPARγ was not apparently changed in Skp2 shRNA-transfected cells compared with the scrambled shRNA-transfected cells, whereas TPA-induced nuclear export of PPARγ was reversed in Skp2 shRNA-transfected cells compared with TPA-treated cells (Figure 5D). These results suggested that down-regulated Skp2 significantly reversed TPA-induced nuclear export of PPARγ in MDA-MB-231 cells.

## 3. Discussion

In this report, we studied the clinical significance and relationship between cytoplasmic localization of PPARγ and Skp2 expression in human breast cancer. Our data revealed that expression of cytoplasmic PPARγ was positively related with Skp2 expression, which is critical to the development and progression of breast cancer. We demonstrated for the first time that Skp2 overexpression provokes cytoplasmic localization of PPARγ upon MEK1-dependent mechanisms in human breast cancer cells.

PPARγ is one of the orphan nuclear receptor superfamily (NRs). It has been previously reported to reside mainly in the nucleus, similar to the vitamin D3 and thyroid hormone receptors [18,19]. Burgermeister *et al*. [7] have recently shown that PPARγ was exported out of the nucleus in response to TPA stimulation, and this shuttle was mediated by the nuclear export signal (NES) in the mitogen-activated protein kinase (MAPK)/extracellular signal-regulated kinase (ERK) kinase 1/2 (MEK1/2). TPA is the most commonly used phorbol ester. It can act as a tumor promoter that induces MMP-9 expression in certain cancer cells [20]. This massive nuclear export reduces the ability of PPARγ to transactivate nuclear target genes and thereby inhibits its genomic function [7]. In this article our results showed that MDA-MB-231(ER-negative) with high invasive capacity displayed mainly cytoplasmic localization of PPARγ. Expression of cytoplasmic PPARγ was increased in poorly differentiated breast carcinoma samples, which was similar to the reports of other research groups [21].

Recently, a growing body of research data has shown that Skp2 plays an oncogenic role and its expression may contribute to the development and progression of human cancers [22]. Overexpression of Skp2 has been reported in several human malignant tumors, including oral squamous cell carcinoma [14,23], ovarian adenocarcinoma [24], lymphoma [25], colorectal carcinomas [26,27], gastric carcinoma [28], soft tissue sarcomas [29], acute myelogenous leukemia [30], and breast cancer [31]. In this study, we found that the expression of cytoplasmic PPARγ was positively related with Skp2 expression (*p* < 0.05) and Skp2 overexpression can provoke nucleocytoplasmic shuttling of PPARγ. This suggests that Skp2 up-regulation plays an important role in reducing the ability of PPARγ to transactivate nuclear target genes. In other words, cytoplasmic PPARγ may lose the ability to reduce cell proliferation and induction of apoptosis in breast cancer cells. However, more studies are required to fully understand the roles of cytoplasmic PPARγ in human breast cancer.

Multiple studies have shown that ERK activities were up-regulated in many human cancers including breast cancer, and elevated ERK activity in human tumors has been correlated with poor prognosis, demonstrating that ERK may play a crucial role in human tumorigenesis [32]. The fact that Skp2 expression was positive correlated with phospho-MAPK/ERK1/2 expression during progression of cervical neoplasia was in accordance with the study of human breast cancer progression [33]. Our results showed that MDA-MB-231 cell lines displayed higher expression level of p-MEK1 than MCF-7 cell lines. Moreover, overexpression Myc-Skp2 resulted in increased MEK1 phosphorylation in MCF-7 cell lines. Importantly, nucleic localization of PPARγ was induced by the MEK inhibitor, (PD98059, U0126), in MDA-MB-231cells. Using RNA interference technology, we found that down-regulated Skp2 reduced the phosphorylation level of MEK1 and significantly reversed TPA-induced nuclear export of PPARγ in MDA-MB-231 cells. It is also possible that the regulation of PPARγ subcellular localization was accomplished by other mechanisms; the mechanisms should be further investigated. These findings suggest that PPARγ is retained in the cytosol upon MEK1-dependent mechanisms in MDA-MB-231 cells.

## 4. Experimental Section

### 4.1. Materials

The antibodies used for immunohistochemistry in this study included: anti-PPARγ (sc-7196, 1:100, Santa Cruz, Biotechnology, Dallas, TX, USA), and anti-Skp2 (sc-74477, 1:100, Santa Cruz, Biotechnology, Dallas, TX, USA). Antibodies for Western blot included: anti-Skp2 (sc-7164, 1:500, Santa Cruz, Biotechnology, Dallas, TX, USA), anti-PPARγ (sc-7196, 1:500, Santa Cruz, Biotechnology, Dallas, TX, USA), anti-pMEK1 (sc-271914, 1:1000, Santa Cruz, Biotechnology, Dallas, TX, USA), anti-MEK1/2 (sc-436, 1:250, Santa Cruz, Biotechnology, Dallas, TX, USA), anti-Lamin B and anti-Tubulin were purchased from Santa Cruz Biotechnology (CA), PD98059, U0126 and tetradecanoyl phorbol acetate (TPA) were from Sigma (St. Louis, MO, USA), β-actin (sc-7196, 1:1000, Santa Cruz, Biotechnology, Dallas, TX, USA).

### 4.2. Cell Culture

Two human breast cancer cell lines: MCF-7(ER-positive), MDA-MB-231(ER-negative), which were gifts from the Department of Oncology, Cancer Hospital of Fudan University were used in this study. All cell lines were maintained in RPMI 1640 (Gibco BRL, Grand Island, NY, USA) supplemented with 10% heat-inactivated fetal calf serum, 2 mM L-glutamine, and 100 U/mL penicillin-streptomycin mixture (Gibco BRL, Grand Island, NY, USA) at 37 °C and 5% CO_2_.

### 4.3. Tissue Samples

Fifty breast cancer and twenty benign breast disease specimens from patients who underwent surgery between 2005 and 2008 at the Department of General Surgery, Affliated Hospital of Nantong University were formalin-fixed and paraffin-embedded for histopathologic diagnosis and immunohistochemical study. Fresh samples were frozen in liquid nitrogen immediately after surgical removal and maintained at −80 °C until use for Western blot analysis. All human tissue was collected using protocols approved by the Ethics Committee of Affiliated Cancer Hospital of Nantong University.

### 4.4. Immunohistochemistry Methods

Serial sections that were 4 μm thick were mounted on glass slides coated with 10% polylysine. Sections were dewaxed in xylene and rehydrated in graded ethanols. Endogenous peroxidase activity was blocked by immersion in 0.3% methanolic peroxide for 40 min. Immunoreactivity was enhanced by microwaving by incubating the tissue sections for 10 min in 0.1 M citrate buffer. Immunostaining was performed using the avidin biotin peroxidase complex method and antigen-antibody reactions were visualized with the chromogen diaminobenzadine.

### 4.5. Immunohistochemical Evaluation

Scoring of immunohistochemical slides was done according to the percentage of tumor cells exhibiting nuclear staining. Two independent pathologists (SP and FM) evaluated the immunostaining. At least ten high-power fields were randomly chosen and at least 300 cells/field was counted in each section. PPARγ positivity was defined as immunoreactive in >5% and negative in no staining or <5% of the cancer cells [21]. A cut-off value of 10% in 10 high-power fields was used to define Skp2 staining [12,34,35]. Immunohistochemical evaluation for ER, PR, p53, Ki-67 and HER-2 were finished by Department of Pathology, Affiliated Hospital of Nantong University.

### 4.6. Expression Plasmid and Transient Transfection

The full-length human Skp2 complementary DNA (cDNA) was amplified by PCR using two oligonucleotides (5′-GCGAATTCATGCACGTATTTTAAACTCC-3′) and (5′-GACTCGAGACTTCATAGACAACTGGGCT-3′) and cloned into pcDNA3.1-myc expression vector at the EcoR I/Xho I. Transfection was performed using lipofectamine 2000 transfection reagent (Invitrogen, Grand Island, NY, USA) according to the manufacture’s protocol with minor modifications. MCF-7 cells were seeded at 2 × 10^5^ cells/mL in a six-well plate 24 h before transfection. 500 μL of transfection mixture containing 8 μg of DNA and 20 μL of lipofectamine 2000 reagent in 450 μL of Opti-MEM (Invitrogen, Grand Island, NY, USA) were added to each well. The cells were harvested 48 h after transfection and used for the experiment. The experiments were repeated at least three times.

### 4.7. Cellular Fractionation

Cells were grown in 10 cm plates to subconfluence. Subsequently, the cells were washed with PBS and scraped into ice-cold buffer H (50 mM-glycerophosphate, pH 7.3, 1.5 mM EGTA, 1 mM EDTA, 1 mM dithiothreitol, 0.1 mM sodium vanadate,1 mM benzamidine, 10 μg/mL aprotinin, 10 μg/mL leupeptin, and 2 μg/mL pepstatin A). The cells were then spun down (12,000× *g*, 5 min), resuspended in 0.1% NP-40, and spun down again. The supernatant containing the cytosolic fraction, was boiled in sample buffer. The pellet containing the nuclei, was resuspended in an extraction buffer (420 mM NaCl, 50 mM-glycerophosphate, 0.5 mM Na_3_VO_4_, 1.5 mM MgCl_2_, 0.2 mM EDTA, 1 mM dithiothreitol, 25% glycerol), and disrupted by sonication (twice for 10 s). The extract was then cleared by centrifugation (12,000× *g*, 5 min), and the supernatant was boiled in sample buffer as the nuclear fraction. Equal amounts of protein of nucleus and cytoplasm were then separated on 10% SDS–polyacrylamide gel electrophoresis.

### 4.8. shRNAs and Transfection

The human Skp2 shRNA expression vector, pGenesil 1.2-EGFP, was constructed. This shRNA targeting the nucleotide residues 5′-AAGGAGATGTCCATGTCCAAG-3′ and 5′-GCCTAAGCTAAATCGAGAGAA-3′ was constructed. MDA-MB-231 cells were seeded the day before transfection using 1640 with 10% FBS without antibiotics. Transient transfection of shRNA vectors and scrambled shRNA vectors were carried out using lipofectamine 2000 and plus reagent in Opti-MEM as suggested by the manufacturer. Cells were incubated with the pGenesil 1.2 vectors and lipofectamine and plus reagent complexes for 4 h at 37 °C. FBS was then added to the cells to achieve a final concentration of 10% in DMEM. Transfected cells were used for subsequent experiments 48 h after transfection.

### 4.9. Western Blot Analysis

Prior to immunoblotting, cells were washed three times with ice-cold PBS, resuspended in 2× lysis buffer (50 mM Tris-HCl, 120 mM NaCl, 0.5% Nonidet P-40, 100 mM NaF, 200 μM Na_3_VO_4_, and protease inhibitor mixture) or frozen tissues were homogenized in lysis buffer (1% NP-40, 50 mmol/L Tris, pH 7.5, 5 mmol/L EDTA, 1% SDS, 1% sodium deoxycholate, 1% Triton X-100, 1 mmol/L PMSF, 10 mg/mL aprotinin, and 1 mg/mL leupeptin) and then incubated for 20 min at 4 °C while rocking. Lysates were cleared by centrifugation (10 min × 12,000 rpm, 4 °C). Western blot procedure was performed as described previously [36]. 50 μg of total protein was resolved by SDS-PAGE and transferred onto polyvinylidene difluoride membranes (Immobilon, Millipore). The membranes were first blocked with 5% nonfat dry milk and then incubated with the primary antibody described above for 2 h at room temperature. After three washes, filters were incubated with horseradish peroxidase-conjugated secondary human anti-mouse or anti-rabbit antibodies (1:1000; pierce) for 1 h at room temperature according to the manufacturer’s instructions. Detection of immunocomplexes was performed with an enhanced chemiluminescence system (NEN Life Science Products, Boston, MA, USA).

### 4.10. Immunofluorescence Microscopy

Cells were fixed on coverslips in 3% paraformaldehyde in PBS, followed by a 10 min permeabilization in 1% Triton X-100 in PBS at 23 °C. Then all cells were blocked with 10% normal serum blocking solution-species the same as the secondary antibody, containing 3% bovine serum albumin (BSA) and 0.1% Triton X-100 and 0.05% Tween-20 two hours at room temperature in order to avoid unspecific staining. Then the coverslips were incubated overnight at 4 °C with primary antibodies against PPARγ. On the following day, Cy3 conjugated (1:1000, Jackson lab) secondary antibodies were added in dark room and incubated for 2–3 h at 4 °C. After three washes in PBS, the cells were mounted onto slides with glycerol containing 1, 4-diazobicyclo (2, 2, 2) octane (DABCO), which were observed under a Leica fluorescence microscope (Leica Microsystems, Wetzlar, Germany).

### 4.11. Statistical Analysis

All computations were carried out using the state 7.0 statistical program. Data were presented as mean ± standard error (se) values of n independent determinations and were triplicated within each experiment. Comparisons were analyzed by using one-way analysis of variance followed by the posteriori Student-Newman-Keul *t*-test. A value of *p* < 0.05 was considered significantly.

## 5. Conclusions

Generally speaking, cytoplasmic localization of PPARγ could become an important therapeutic target in breast cancer for several reasons. First, the expression of cytoplasmic PPARγ was positively related with Skp2 expression (*p* < 0.05) and correlated significantly with estrogen receptor (*p* = 0.026), pathological grade (*p* = 0.029). However, hormone therapy has little effect on ER-negative tumors. Second, Skp2 induced an export of PPARγ as observed above, and this reduced the amount of active PPARγ in the nucleus and thereby inhibited its genomic function. Third, MDA-MB-231(ER-negative) displayed mainly cytoplasmic localization of PPARγ. Finally, the MEK-ERK signaling pathway has been shown to play a critical role in the survival and growth of breast cancer cells. Thus, it may be a new idea for the ER-negative breast cancer prevention and treatment to induce PPARγ nuclear export. Next, we are planning to study the correlation between cytoplasmic PPARγ expression and patient survival further, and develop a MEK-ERK pathway signature in breast cancer cells that could be used for patient selection for treatment with a MEK inhibitor.

## Figures and Tables

**Table 1:** The expression of cytoplasmic PPARγ in relation to the clinical and pathological characteristics of patients.

Criteria	Levels of cytoplasmic PPARγ		

	Low	High	*p* value a
ER			
0	5	8	0.026 *
1	27	10	

PR			
0	9	10	0.055
1	23	8	

HER-2			
Negative	11	6	0.941
Positive	21	12	

p53			
Negative	13	11	0.164
Positive	19	7	

TNM			
I	9	4	0.580
II	18	9	
III	5	5	

Grade			
1	10	1	0.029 *
2	18	10	
3	4	7	

Ki-67			
Negative (<19)	8	7	0.304
Positive (≥19)	24	11	

Skp2			
Low (<10)	25	0	0.000 *
High (≥10)	7	18	

a Statistical analyses were performed by the Pearson *x*^2^ test;

* *p* < 0.05 is considered significant.
